# Own Race Eye-Gaze Bias for All Emotional Faces but Accuracy Bias Only for Sad Expressions

**DOI:** 10.3389/fnins.2022.852484

**Published:** 2022-05-12

**Authors:** Xiaole Ma, Meina Fu, Xiaolu Zhang, Xinwei Song, Benjamin Becker, Renjing Wu, Xiaolei Xu, Zhao Gao, Keith Kendrick, Weihua Zhao

**Affiliations:** ^1^Key Laboratory for NeuroInformation of Ministry of Education, Center for Information in Medicine, The Clinical Hospital of Chengdu Brain Science Institute, University of Electronic Science and Technology of China, Chengdu, China; ^2^School of Education Science, Shanxi University, Taiyuan, China; ^3^School of Foreign Languages, University of Electronic Science and Technology of China, Chengdu, China

**Keywords:** own-race bias, other-race bias, gaze patterns, facial expressions, eye tracking

## Abstract

Own race faces tend to be recognized more accurately than those of other less familiar races, however, findings to date have been inconclusive. The present study aimed to determine whether Chinese exhibit different recognition accuracy and eye gaze patterns for Asian (own-race) and White (other-race) facial expressions (neutral, happiness, sadness, anger, disgust, fear). A total of 89 healthy Chinese adults viewed Asian and White facial expressions while undergoing eye-tracking and were subsequently required to identify expressions and rate their intensity and effect on arousal. Results revealed that subjects recognized sad expressions in Asian faces better than in White ones. On the other hand, recognition accuracy was higher for White neutral, happy, fearful, and disgusted expressions although this may have been due to subjects more often misclassifying these Asian expressions as sadness. Moreover, subjects viewed the eyes of emotional expressions longer in Asian compared to White faces and the nose of sad ones, especially during the late phase of presentation, whereas pupil sizes, indicative of cognitive load and arousal, were smaller. Eye-gaze patterns were not, however, associated with recognition accuracy. Overall, findings demonstrate an own-race bias in Chinese for identifying sad expressions and more generally across emotional expressions in terms of viewing the eye region of emotional faces for longer and with reduced pupil size. Interestingly, subjects were significantly more likely to miss-identify Asian faces as sad resulting in an apparent other-race bias for recognizing neutral, happy, fearful, and disgusted expressions.

## Introduction

It is of critical importance for successful social interaction to precisely interpret facial emotional expressions which reflect a person’s current affective state, motives and intentions (Addington et al., 2006). Convergent evidence has demonstrated that emotional expression processing and recognition depends largely on viewing distinctive features, particularly the eyes, and also specific patterns of viewing (see Beaudry et al., 2014). Although the presence of a face triggers a universal, biologically determined information extraction pattern (Henderson et al., 2005; Blais et al., 2008), there are some factors which may make differences in emotion perception and emotion judgments, such as culture (Jack et al., 2009; Stanley et al., 2013), own-race bias/in-group bias (Meissner and Brigham, 2001). Cultural differences are evidenced by different gaze patterns with Asian observers more persistently fixating the eye region during processing of face expressions whereas Western observers distribute their fixations more evenly across the face (Jack et al., 2009). A number of studies reported an emotional recognition bias for own-race faces, however, specific facial features have not been systematically examined using eye-tracking to underly perceptual and cognitive mechanisms.

An own-race bias suggesting that faces are recognized and identified more accurately by members of the same race than another one has been repeatedly reported by several studies (e.g., Meissner and Brigham, 2001; Elfenbein and Ambady, 2002, 2003). For example, accuracy was higher when emotions were both expressed and recognized by members of the same national, ethnic, or regional group, suggesting an in-group advantage (Elfenbein and Ambady, 2002). However, scanning pattern differences (i.e., fixation, gaze, and pupil size) which may contribute to own-race bias remain unclear. Prior eye-tracking studies have revealed a systematic gaze bias predominantly toward the eyes, but also to the nose and mouth regions which are thought to convey substantial social information for interpersonal communication (e.g., Hadjikhani et al., 2008) and contribute to recognition of the underlying emotion (Schyns et al., 2002). Specifically, the eye region is focused on more in sad and angry facial expressions, whereas the mouth for happy and disgusted expressions. In addition, recognition of fear and surprise expressions depends on all three facial regions (Eisenbarth and Alpers, 2011; Bombari et al., 2013). It has been reported that adult Asians tend to fixate on the central regions of faces (i.e., nose) when scanning both same- and other-race faces, whereas the White produce a scattered triangular scanning pattern (Blais et al., 2008). On the contrary, several studies have reported that Chinese adults fixate more on the eye regions of White than Chinese faces and more on the nose region of Chinese faces in face recognition tasks using neutral expressions (Fu et al., 2012; Hu et al., 2014). Thus, differences in fixation durations or gaze patterns between Asian and White faces during facial expression processing are inconsistent (Nusseck et al., 2008; Beaudry et al., 2014; Schurgin et al., 2014; Calvo et al., 2018).

In addition, pupil size is an objective and reliable index for both affective and cognitive information processing. This is driven by two muscles (i.e., “dilatator” and “sphincter” muscles) innervated by the sympathetic and parasympathetic branches of the autonomic nervous system (Granholm and Steinhauer, 2004). Peak dilation has been associated with the demands for emotional valence identification (Prehn et al., 2011) and pupil size also conveys information on emotional arousal (Bradley et al., 2008) and approach behavior (Laeng and Falkenberg, 2007). Adults exhibit larger pupil diameters when processing emotional expressions compared to neutral ones (Bradley et al., 2008). However, little is known about pupil size patterns when processing own-race vs. other-race expressions during natural viewing states. In addition, durations of first fixations to specific regions were shown to be related to hit rate of facial expression recognition, supporting the conclusion that first fixation duration is important for accurate processing of facial expression information (Hall et al., 2010).

Against this background, in order to better establish both similarities and differences in the processing of Asian and White expressions, we monitored the eye movements of Chinese observers including fixation pattern, first gaze and pupil size as measurements during a free-viewing task using both Asian (own-race) and White (other-race) faces. This was followed by a behavioral evaluation task where observers were instructed to perform a six-alternative forced-choice facial expression categorization (i.e., neutral, happiness, sadness, anger, disgust, and fear) and evaluate the expression in terms of intensity and arousal (1–9 points), respectively. Overall, we hypothesized that participants’ proportional fixation duration on specific regions of Chinese faces would be different from those of White faces across facial expressions and that pupil sizes would be larger when processing White than Asian faces indicating the presence of an own-race bias during facial expression processing.

## Materials and Methods

### Participants

One hundred and one healthy college students were recruited in the present study. Exclusion criteria were any previous or current neurological or psychiatric disorders, as well as current or regular use of any psychotropic substances. All participants had normal or corrected to normal vision. Two participants failed to complete the whole procedure and nine participants were excluded from data analysis due to technical problems leaving a total of 89 participants (male = 44; Mean age ± SD = 22.15 ± 2.03 years). *A priori* power calculation using G^∗^Power (version 3.1.9.2) showed that 66 participants would be sufficient to achieve 90% power for a medium effect size of 0.4 at α = 0.05 with repeated ANOVA (within factors: expression, face race, and region). A *post hoc* analysis was also conducted and found the power reached 96.8% with our final sample size included in the analysis (*n* = 89). All participants were Han Chinese and instructions and other study materials were provided in Chinese. All participants provided written informed consent before the experiment and were paid monetary compensation for participation. This study was approved by the local ethics committee of the University of Electronic Science and Technology of China and was in accordance with the latest revision of the Declaration of Helsinki. All data is available at the following link https://osf.io/uxtbp.

### Stimuli and Procedure

Professional Chinese actors (*n* = 30, 15 males, aged between 18 and 30 years old) were recruited for producing the facial expression stimuli. The actors were instructed not to wear any particularly distinctive items (scarf, jewelry, make-up, glasses, etc.) and tie up their hair. The actors were required to look straight ahead and pose specific emotional expressions (happy, sad, angry, disgusted, and fearful) naturally and slowly with direct gaze and frontal view. The dynamic facial expressions were recorded using a professional camera (Nikon D7200) and subsequently static expressions were captured with a frame capturing software. Pictorial stimuli were divided into two and evaluated online by two independent samples (*n* = 150 and *n* = 145). Subjects in each group were required to rate expression specificity (“To what extent does this face express neutral/happy/sad/angry/disgusted/fearful expression, respectively”; (1-point scale, 0 = extremely low; 10 = extremely high). Based on these ratings, a final Asian database of 96 face stimuli from 16 actors (8 males) with 6 different facial expressions (neutral, anger, disgust, fear, happiness, and sadness) was selected. Sixteen White actors (8 males) with corresponding expressions selected from the NimStim Set of Facial Expressions were additionally included (Tottenham et al., 2009). In order to match stimuli from the two different datasets, all the stimuli were modified to have the same head position and resolution and rescaled to 800 × 800 pixels. All stimuli were overlaid with an identical ellipse mask to cover hair, ears etc. (against a black background). Stimulus manipulation has also been conducted through matching color attributes for Asian or White complexion, respectively, and all the images were matched in overall luminance using Photoshop software (see Table 1). The position of the eyes and the mouth were aligned between stimuli for the eye-tracking acquisition. Considering reported differences between mouth open as opposed to closed for recognition of sad, fear and happy faces in the NimStim dataset (Tottenham et al., 2009), we also matched the number of open mouth stimuli for sad, fear, and happy across the two face expression sets.

**TABLE 1:** Stimuli color matching across two datasets.

	RGB		Luminance	
	Asian	White	Asian	White
Anger	135.6	139.8	139.5	140.7
Disgust	135.4	138.8	139.4	139.7
Fear	135.6	137.8	139.6	138.8
Happiness	135.4	138.3	139.4	139.1
Neutral	136.4	138.8	140.4	139.8
Sadness	135.6	138.6	139.8	139.6
				

Prior to the formal study, an independent sample (*n* = 20, 10 females) were recruited to behavioral identify expression category (recognition accuracy) for Asian (*n* = 96) and White (*n* = 96) expressions and expression intensity ratings. Subjects were asked to choose the word which best described the facial expression for the person with no response time constraint (1 = Neutral, 2 = Happiness, 3 = Sad, 4 = Anger, 5 = Disgust, and 6 = Fear) and intensity ratings (1–9 points). Results indicated no accuracy/intensity difference between Asian and White faces for each expression (*p*s*_*bonf*_* > 0.05, Bonferroni correction), indicating that Asian and White face-expression stimuli from the two datasets were reasonably matched.

The formal task comprised two components: (1) Subjects simply viewed the face stimuli with concomitant eye-tracking; (2) Subjects next viewed the face stimuli without eye tracking while providing behavioral ratings. During the free-viewing task with eye-tracking, stimuli were presented in the center of a 17-inch monitor at a resolution of 1,024 × 768 pixels (60 Hz) using E-prime 2.0 (Psychology Software Tools, Inc.). A chin rest was used to standardize the distance from the screen to the eyes (57 cm away and centrally positioned relative to the monitor) and to minimize head movements. The eye gaze data during face presentation was acquired using an EyeLink 1000 Plus system (SR Research, Ottawa, Canada) in monocular mode (right eye) at a sampling rate of 2,000 Hz in a dimly illuminated room. A nine-point calibration was conducted before each block (implemented in the EyeLink application programming interface) to establish optimal calibration (drift correction < 1 of visual angle). A total of 192 images were randomly presented in one block of Asian faces (96 trials, male = 48 trials, 8.8 min) and one block of White faces (96 trials, male = 48 trials, 8.8 min) with a break (30–60 s) between them, and a counterbalanced order across participants. Each block consisted of both emotional (Happy, Sad, Angry, Disgusted, Fearful) and neutral expression faces. Each trial started with a white cross for 2,000–3,000 ms followed by a face image for 3,000 ms. Participants were instructed to fixate the cross and to watch each facial expression image freely, respectively. The position of the cross was equivalent to the nasion of the face and was not included in any areas of interest.

In the second part of the task the subjects viewed all faces again but in a different randomized order. Subjects were required to make expression category judgments (recognition accuracy) and provide intensity (1 = slight, 5 = moderate, and 9 = strong) and arousal (1 = low, 5 = moderate, and 9 = high) ratings using a 9-point Likert scale. Subjects were required to respond as fast and as accurately as possible during the expression recognition although there was no time limit for responses on each image. Accuracy for expression category and response times (RTs), as well as rating scores were collected.

### Areas of Interest Definition and Statistical Analysis

**FIGURE 1:**
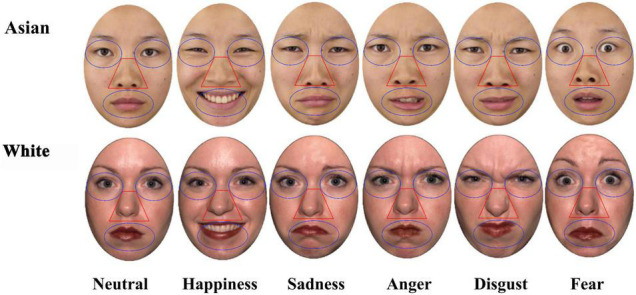
Areas of interest (AOIs, i.e., eyes, nose, and mouth) for the different face expressions (Neutral, Happiness, Sadness, Anger, Disgust, and Fear) for Asian and White example images.

Based on the previous literature (Hu et al., 2017), eyes, nose and mouth regions were created as areas of interest (AOI; see Figure 1) to investigate facial expression processing patterns with no overlap between AOIs (left eye = 10,210 Pixels; right eye = 10,210 Pixels; nose = 10,017 Pixels; mouth = 14,137 Pixels). An average for the left and right eyes was used for subsequent analysis.

The raw eye tracking data was initially exported and processed using the EyeLink DataViewer 3.1 (SR Research, Mississauga, Ontario, Canada) and subsequent data was analyzed with SPSS 24.0 software (SPSS Inc., Chicago, Illinois, United States). Indices measured included fixation duration proportion (%), first fixation duration and pupil size on different regions (eyes, nose, and mouth). Fixation duration proportion was calculated by dividing the sum of the fixation duration on each AOI (eyes, nose, or mouth) by the total fixation time on the whole image. First fixation duration was the duration of the first fixation on each AOI. Average fixation pupil size was extracted from the interest area report of average pupil size across all fixations in the interest area.

Considering that some studies have reported that women identify facial expressions better than men (Hall and Matsumoto, 2004; Hall et al., 2010), we firstly determined that there were no sex differences in behavioral and eye tracking data using independent *t*-tests with Bonferroni correction. Two-way repeated ANOVAs with face expression (Neutral, Happiness, Sadness, Anger, Disgust, Fear) and face race (Asian vs. White) as within-subject factors were then performed for behavioral measurements (recognition accuracy, intensity, and arousal ratings).

It has been confirmed that pupils size changed when people perform more difficult cognitive operations. We conducted pupil dilation analyses where in participants’ eyes were focused on the same AOIs across conditions because we aim to explore the pupil size pattern during affective and cognitive information processing on specific features. Additionally, the stimuli had variations across stimulus displays due to presentation of different visual images (Asian and White faces) although well controlled, the pupil analyses were performed on featural AOIs instead of the whole face, which help minimize the influence of pupils’ visual reflexes (Goldinger et al., 2009). Thus, three-way repeated ANOVAs with region (eye, nose, mouth), face expression and face race as within-subject factors were conducted on each eye-tracking measurement (fixation duration proportion, first fixation duration, and pupil size). In addition, to further explore the time course of fixation on eyes during face displays, the 3,000 ms expression display duration was divided into 3 consecutive intervals of 1,000 ms (i.e., early phase: 0–1,000 ms, middle phase: 1,000–2,000 ms, late phase: 2,000–3,000 ms) and the proportion of gaze for each interval was extracted and analyzed. Pearson correlations were conducted to explore the relationship between recognition accuracy and eye gaze and pupil size data with Bonferroni correction. The assumption of sphericity was assessed with Mauchly’s test and the Greenhouse-Geisser correction for non-sphericity applied when necessary. Bonferroni correction was used for all *post hoc* pairwise comparisons. Partial η^2^ (for *F*-test) or Cohen’s *d* (for *t*-test) were calculated as measures of effect size. Tests employed two-tailed *p*-values, with *p* < 0.05 considered significant.

## Results

There were no participant sex differences in terms of behavioral measurements as well as eye tracking data (all *p*s > 0.23), thus, participant sex was not further accounted for in the following analyses.

### Behavioral Results

#### Expression Recognition Accuracy

**FIGURE 2:**
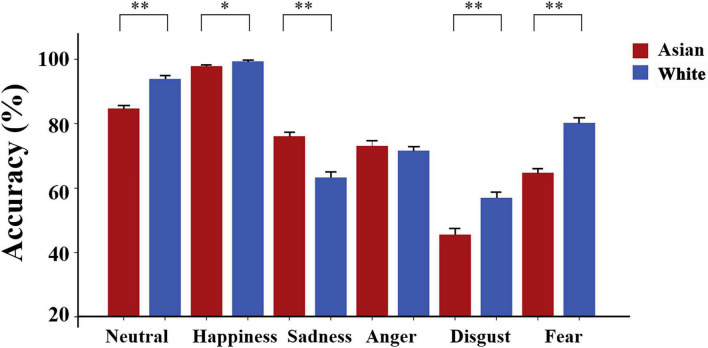
Differences in recognition accuracy for Asian and White expressions. Histograms show mean ± SEM% accuracy. ^∗^*p* < 0.05 and ^∗∗^*p* < 0.01 for Asian vs. White faces.

A two-way repeated measured ANOVA with within-subject factors of expression and face race revealed main effects of expression [*F*_(5, 440)_ = 179.57, *p* < 0.001, η*_*p*_*^2^ = 0.67, Happiness > Neutral > (Fear∼ Anger∼ Sadness) > Disgust], and face race [*F*_(1, 88)_ = 32.61, *p* < 0.001, 95% CI for difference = (−5.18, −2.51), η*_*p*_*^2^ = 0.27] reflecting higher expression recognition accuracy for White relative to Asian faces. There was also an interaction between expression and face race [*F*_(5, 440)_ = 39.37, *p* < 0.001, η*_*p*_*^2^ = 0.31] suggesting that participants exhibited a higher accuracy for recognizing White faces compared with Asian faces for neutral [*t*_(88)_ = −6.71, *p*_*bonf*_ < 0.001, 95% CI for difference = (−11.95, −6.49), Cohen’s *d* = 0.71], happy [*t*_(88)_ = −2.91, *p*_*bonf*_ = 0.005, 95% CI for difference = (−2.51, −0.47), Cohen’s *d* = 0.31], disgusted [*t*_(88)_ = −5.59, *p*_*bonf*_ < 0.001, 95% CI for difference = (−15.42, −7.37), Cohen’s *d* = 0.59] and fearful [*t*_(88)_ = −8.51, *p*_*bonf*_ < 0.001, 95% CI for difference = (−19.07, −11.85), Cohen’s *d* = 0.90] expressions but the opposite pattern for sad expressions [*t*_(88)_ = 6.26, *p*_*bonf*_ < 0.001, 95% CI for difference = (8.78, 16.94), Cohen’s *d* = 0.66] and no difference for angry expressions [*t*_(88)_ = 1.02, *p* = 0.310, 95% CI for difference = (−1.53, 4.76), see Figure 2]. Thus, we only found evidence for an own-race bias in recognizing sad emotional expression faces but an other-race bias for neutral, happy, disgusted and fearful expression faces. There were no significant differences in response times for judging all types of expressions between Asian and White faces (all *ps* > 0.08) or when only correct responses were included (all *p*s > 0.14). Interestingly, when we analyzed the patterns of errors shown by subjects for recognizing neutral faces, they were significantly more likely to erroneously classify Chinese faces as having a sad expression than for White faces [M_Asian_ = 9.35% ± 8.7; M_White_ = 1.97% ± 5.46, *t* = 7.37, *p* < 0.001, 95% CI for difference = (5.39, 9.37), Cohen’s *d* = 0.78], further supporting the own race bias toward recognition of sad expressions. This was not the case for angry faces where there was no significant race bias (M_Asian_ = 4.00% ± 6.27; M_White_ = 4.42% ± 6.40, *t* = −0.64, *p* = 0.525) indicating a highly emotion-specific effect. Importantly, the overall% of trials where subjects made sadness miss-classification errors for expressions with a significant accuracy advantage for identifying the White faces (i.e., Neutral, Happy, Fearful, Disgusted) was significantly greater in Asian than White faces [M_Asian_ = 11.66% ± 5.91; M_White_ = 6.65% ± 4.39, *t* = 8.76, *p* < 0.001, 95% CI for difference = (3.87, 6.14), Cohen’s *d* = 0.93].

#### Intensity Ratings

A two-way repeated ANOVA with the within-subject factors expression and face race revealed main effects of expression [*F*_(5, 440)_ = 412.08, *p* < 0.001, η*_*p*_*^2^ = 0.82] and face race [*F*_(1, 88)_ = 8.68, *p* = 0.004, 95% CI for difference = (−0.28, −0.06), η*_*p*_*^2^ = 0.09], reflecting higher intensity ratings for White relative to Asian faces. There was also an interaction between expression and face race [*F*_(5, 440)_ = 15.15, *p* < 0.001, η*_*p*_*^2^ = 0.15]. Pairwise comparisons with Bonferroni correction revealed that participants had a higher intensity rating for White faces relative to Asian angry [*t*_(88)_ = −3.52, *p*_*bonf*_ < 0.001, 95% CI for difference = (−0.45, −0.13), Cohen’s *d* = 0.37], disgusted [*t*_(88)_ = −4.56, *p*_*bonf*_ < 0.001, 95% CI for difference = (−0.54, −0.21), Cohen’s *d* = 0.48] and fearful expressions [*t*_(88)_ = −4.57, *p*_*bonf*_ < 0.001, 95% CI for difference = (−0.54, −0.22), Cohen’s *d* = 0.49] but the opposite for neutral faces [*t*_(88)_ = 3.30, *p*_*bonf*_ = 0.001, 95% CI for difference = (0.10, 0.38), Cohen’s *d* = 0.35]. No significant differences were found for happy [*t*_(88)_ = −1.02, *p* = 0.313, 95% CI for difference = (−0.22, 0.07)] or sad faces [*t*_(88)_ = −1.64, *p* = 0.105, 95% CI for difference = (−0.31, 0.03)].

#### Arousal Ratings

A two-way repeated ANOVA with the within-subject factors expression and face race revealed main effects of expression [*F*_(5, 440)_ = 236.26, *p* < 0.001, η*_*p*_*^2^ = 0.73, Happiness > (Anger∼ Disgust ∼ Fear) > Sadness > Neutral], but not face race [*F*_(1, 88)_ = 3.55, *p* = 0.06, 95% CI for difference = (−0.29, 0.01)]. There was an interaction between expression and face race [*F*_(5, 440)_ = 6.14, *p* < 0.001, η*_*p*_*^2^ = 0.07]. Pairwise comparisons with Bonferroni correction revealed that subjects reported higher arousal for White compared with Asian faces for angry [*t*_(88)_ = −2.29, *p*_*bonf*_ = 0.024, 95% CI for difference = (−0.41, −0.03), Cohen’s *d* = 0.24], disgusted [*t*_(88)_ = −3.30, *p*_*bonf*_ = 0.001, 95% CI for difference = (−0.52, −0.13), Cohen’s *d* = 0.35] and fearful expressions [*t*_(88)_ = −2.21, *p*_*bonf*_ = 0.030, 95% CI for difference = (−0.43, −0.02), Cohen’s *d* = 0.23] but not neutral [*t*_(88)_ = 1.24, *p* = 0.218, 95% CI for difference = (−0.06, 0.27)], happy [*t*_(88)_ = −1.45, *p* = 0.151, 95% CI for difference = (−0.30, 0.05)] and sad expressions [*t*_(88)_ = −0.56, *p* = 0.579, 95% CI for difference = (−0.23, 0.13)].

### Eye-Tracking Results

#### Fixation Duration Proportions

**FIGURE 3:**
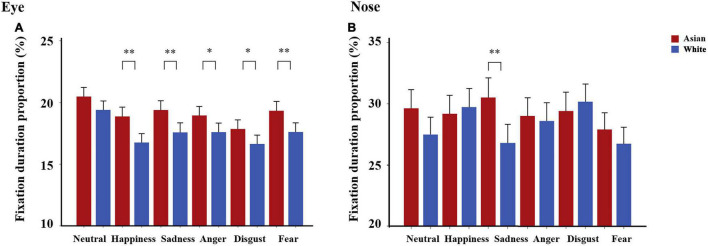
Differences in fixation duration proportion (%) of Asian and White facial expressions. Fixation duration proportions for each facial expression between Asian and White for **(A)** eye and **(B)** nose were shown. Bars represent mean fixation duration proportion across trials. Error bars represent standard errors of the means. ^∗^*p* < 0.05, ^∗∗^*p* < 0.01 for Asian vs. White.

**FIGURE 4:**
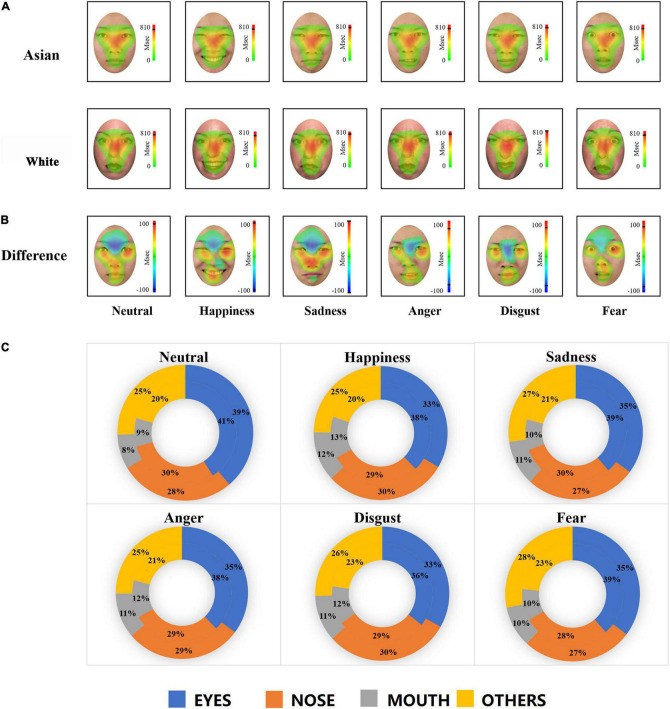
Heat maps and doughnut diagrams of fixation distributions on Asian and White facial expressions. **(A)** Heat map of fixation distributions for each facial expression in Asian (top) and White (bottom) faces. Color-coded distributions represent the duration of fixations across face regions, with red indicating higher fixation proportions. **(B)** Heat map of differences between Asian faces and White faces for each expression. Color-coded distributions represent the different duration of fixations across face regions, with red indicating the regions where Asian faces were fixated more than White faces and blue indicating the opposite pattern. A black line is drawn on the heat map scale to indicate the actual maximum activation of the heat map. A default low activity cut-off value (10%) is used on both sides of the scale so that no coloring is applied when the difference is small. **(C)** Doughnut of fixation distributions including eyes (total of the left eye and right eye), nose, mouth, and rest of the facial regions across expression and face race. The outer lane indicates the pattern for White faces and the inner lane for Asian faces. *p* (eyes) = *p* (left eye) + *p* (right eye); *p* (others) = 1-*p* (eyes)-*p* (nose)-*p* (mouth).

For fixation duration proportion (%), a three-way ANOVA with within-subject factors of expression, face race and AOI region (eye, nose, mouth) was conducted. We observed significant main effects of region [*F*_(2, 176)_ = 77.51, *p* < 0.001, η*_*p*_*^2^ = 0.47] due to the mouth region being viewed less than the eyes and nose regions (*p*s < 0.001), main effect of expression [*F*_(5, 440)_ = 23.86, *p* < 0.001, η*_*p*_*^2^ = 0.21], indicating longer viewing durations on the AOIs for happy faces and shorter for fearful faces, and face race [*F*_(1, 88)_ = 9.37, *p* = 0.003, 95% CI for difference = (0.38, 1.77), η*_*p*_*^2^ = 0.10] indicating that subjects viewed all three AOIs of Asian faces more than White faces. There was a significant region × expression interaction [*F*_(10, 880)_ = 15.33, *p* < 0.001, η*_*p*_*^2^ = 0.15] as well as expression × face race interaction [*F*_(5, 440)_ = 2.51, *p* = 0.029, η*_*p*_*^2^ = 0.03], but more importantly there was a significant three-way region × expression × face race interaction [*F*_(10, 880)_ = 5.89, *p* < 0.001 η*_*p*_*^2^ = 0.06]. Pairwise comparisons with Bonferroni correction revealed that: (a) the eye region was viewed more in Asian relative to White faces for all emotional expressions [*t*_(88)_ > 2.01, *ps_*bonf*_* < 0.048, Cohen’s *d* > 0.21] but not neutral ones [*t*_(88)_ = 1.63, *p* = 0.11, 95% CI for difference = (−0.23, 2.38); see Figure 3A]; (b) the nose region was viewed more in Asian relative to White faces for sad [*t*_(88)_ = 3.01, *p*_*bonf*_ = 0.003, 95% CI for difference = (1.26, 6.15), Cohen’s *d* = 0.32] but not for other expressions (all *ps* > 0.06, see Figure 3B); (c) no significant differences in fixation duration proportion were found for the mouth region (all *ps* > 0.07). In addition, we also found similar patterns when only including correct responses with a three-way region × expression × face race interaction [*F*_(10, 880)_ = 3.94, *p* < 0.001, η*_*p*_*^2^ = 0.04]. *Post-hoc* tests with Bonferroni correction showed that (a) the *eye* region was fixated more in Asian than White faces for all emotional expressions [*t*_(88)_ > 2.04, *ps_*bonf*_* < 0.044, Cohen’s *d* > 0.21] but not neutral expressions [*t*_(88)_ = 1.50, *p* = 0.138]; (b) the nose region was viewed longer for Asian compared to White sad expressions [*t*_(88)_ = 2.85, *p*_*bonf*_ = 0.005, Cohen’s *d* = 0.30] but not for other expressions [*t*_(88)_ < 1.95, *ps* > 0.05]; (c) the mouth region was comparable between the two races [*t*_(88)_ < 1.81, *ps* > 0.07]. Heat maps showing fixation distributions for Asian and White faces as well as differences between them for each expression including eyes, nose, mouth, and rest of the facial regions are shown in Figure 4.

**FIGURE 5:**
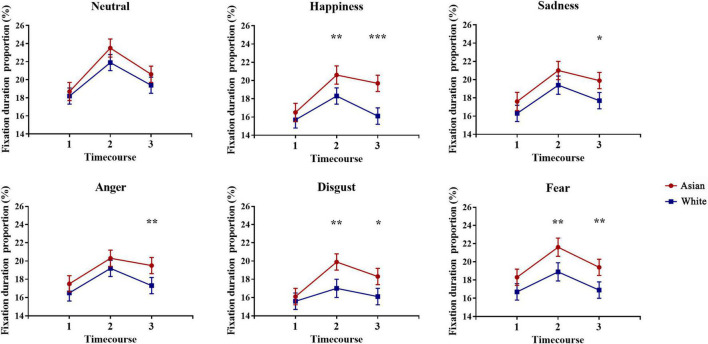
Time course of fixation duration proportions on the eye region of Asian and White facial expressions. Duration of each emotional expression (Neutral, Happiness, Sadness, Anger, Disgust, and Fear) processing (i.e., 3,000 ms) was divided into three phases [(1) early: 0–1,000 ms, (2) middle: 1,000–2,000 ms, (3) late: 2,000–3,000 ms]. Lines represent mean fixation duration proportion across races (red-Asian; blue-White) at different time bins. Error bars represent standard errors of the means. *p < 0.05, **p < 0.01, ***p < 0.001 for Asian vs. White.

Considering the crucial role of eye region in expression processing, to further identify gaze patterns toward the eye region for own-race and other-race faces during expression processing, we divided the duration of emotion processing (i.e., 3,000 ms) into three consecutive phases (early phase: 0–1,000 ms, middle phase: 1,000–2,000 ms, late phase: 2,000–3,000ms). A repeated two-way ANOVA including 2 (Face race: Asian, White) × 3 (Time bins: 0–1,000 ms, 1,000–2,000 ms, 2,000–3,000 ms) factors was performed on fixation duration proportion on the eye region for each expression. Analyses revealed significant main effects of face race for all emotional expressions [Happiness: *F*_(1, 88)_ = 10.99, *p* = 0.001, 95% CI for difference = (0.89, 3.54), η*_*p*_*^2^ = 0.11; Sadness: *F*_(1, 88)_ = 5.72, *p* = 0.019, 95% CI for difference = (0.29, 3.15), η*_*p*_*^2^ = 0.06; Anger*: F*_(1, 88)_ = 5.13, *p* = 0.026, 95% CI for difference = (0.18, 2.77), η*_*p*_*^2^ = 0.06; Disgust: *F*_(1, 88)_ = 7.54, *p* = 0.007, 95% CI for difference = (0.50, 3.14), η*_*p*_*^2^ = 0.08; Fear*: F*_(1, 88)_ = 11.93, *p* = 0.001, 95% CI for difference = (0.95,3.51), η*_*p*_*^2^ = 0.12] indicating a longer fixation duration on the eye region for Asian emotional expressions compared with White faces, and main effects of time bins for all expressions (all *p*s < 0.01) as well as face race × time bins interactions for happy [*F*_(2, 176)_ = 4.80, *p* = 0.009, η*_*p*_*^2^ = 0.05] and disgusted [*F*_(2, 176)_ = 4.11, *p* = 0.018, η_*p*_^2^ = 0.05] expressions. *Post hoc* multiple comparison contrasts showed differences between Asian and White faces for happy and disgusted expressions at 1,000–2,000 ms [Happiness: *t*_(88)_ = 2.93, *p*_*bonf*_ = 0.004, 95% CI for difference = (0.76, 3.99), Cohen’s *d* = 0.31; Disgust: *t*_(88)_ = 3.38, *p*_*bonf*_ = 0.001, 95% CI for difference = (1.19, 4.58), Cohen’s *d* = 0.36] and 2,000–3,000 ms [Happiness: *t*_(88)_ = 4.02, *p*_*bonf*_ < 0.001, 95% CI for difference = (1.78, 5.27), Cohen’s *d* = 0.43; Disgust: *t*_(88)_ = 2.62, *p*_*bonf*_ = 0.010, 95% CI for difference = (0.52, 3.77), Cohen’s *d* = 0.28]. Further exploratory analysis also showed race differences for sad [2,000–3,000 ms: *t*_(88)_ = 2.62, *p*_*bonf*_ = 0.010, 95% CI for difference = (0.54, 3.90), Cohen’s *d* = 0.28]; angry [2,000–3,000 ms: *t*_(88)_ = 2.74, *p*_*bonf*_ = 0.007, 95% CI for difference = (0.61, 3.86), Cohen’s *d* = 0.29]; fearful [1,000–2,000 ms: *t*_(88)_ = 3.36, *p*_*bonf*_ = 0.001, 95% CI for difference = (1.10, 4.28), Cohen’s *d* = 0.36; 2,000–3,000 ms: *t*_(88)_ = 3.18, *p*_*bonf*_ = 0.002, 95% CI for difference = (0.93, 4.03), Cohen’s *d* = 0.34] expressions. The significant differences across face expressions within each time interval are shown in Figure 5.

#### First Fixation Duration

For the first fixation duration, a 3 (AOI: eye, nose, mouth) × 6 (Expression: Neutral, Happiness, Sadness, Anger, Disgust, Fear) × 2 (Face race: White vs. Asian) repeated ANOVA was conducted and we observed a main effect of region [*F*_(2, 176)_ = 7.66, *p* = 0.002, η*_*p*_*^2^ = 0.08], with first fixations being toward nose mostly. We also found a significant interaction between region × expression [*F*_(10, 880)_ = 2.53, *p* = 0.017, η*_*p*_*^2^ = 0.03] indicating longer duration of first fixation on the mouth for happy faces compared with sad faces. There were no significant main or interaction effects involving face race, however (all *p*s > 0.07).

#### Average Fixation Pupil Size

For fixation pupil size, we observed a main effect of region [*F*_(2, 176)_ = 121.30, *p* < 0.001, η*_*p*_*^2^ = 0.58, nose > eyes > mouth] and face race [*F*_(1, 88)_ = 21.70, *p* < 0.001, 95% CI for difference = (−154.76, −62.20), η*_*p*_*^2^ = 0.20], indicating larger fixation pupil size for White expressions than Asian ones, and a significant region × face race interaction [*F*_(2_,_176)_ = 8.30, *p* < 0.001, η*_*p*_*^2^ = 0.09]. Pairwise comparisons with Bonferroni adjustment revealed that pupil size was smaller when subjects viewed Asian relative to White faces for eyes (*p*_*bonf*_ < 0.001), nose (*p*_*bonf*_ = 0.015), and mouth regions (*p*_*bonf*_ < 0.001).

### Correlations

To identify the relationship between expression recognition performance and fixation indexes, a bivariate correlation analysis was conducted. However, there were no correlations between recognition accuracy and fixation duration proportions on different AOIs or pupil size for either Asian or White faces (all *p*s > 0.22) which would pass correction for multiple comparisons.

In view of the possibility that greater face-recognition accuracy observed for White faces (neutral/happiness/disgust/fear) might have been contributed by higher intensity and arousal ratings compared with Asian faces we also ran correlation analyses between them. There were, however, no significant correlations (all *ps* > 0.42) suggesting that neither intensity nor arousal ratings strongly influenced accuracy of face emotion expression judgments for White faces.

## Discussion

Our present study investigated whether Chinese observers recognize emotional expressions of Asian (own-race) and White (other-race) faces differently using behavioral measurements in combination with eye-tracking. We found an own-race bias advantage in recognizing sad expression faces although intensity and arousal ratings were comparable. On the contrary, subjects exhibited a higher accuracy for recognizing the majority of expressions in White faces (i.e., neutral, happy, fearful, and disgusted), however, this may have been as a result of error patterns revealing that subjects were significantly more likely to misclassify these four expressions as sad in Asian faces. There were no race differences observed for angry expression faces. For eye-tracking measures subjects spent a greater proportion of time viewing the eye-regions of Asian emotional but not neutral faces compared with White ones, particularly during the late phase of presentation, and also the nose region of sad expression faces. Additionally, subjects’ pupil size was smaller when viewing the eyes, mouth, and nose regions of Asian than White emotional faces. Thus overall, our findings indicated the presence of an own-race bias only for recognition of sad expression faces while in terms of greater time spent viewing the eyes and smaller pupil size there was an own-race bias for all emotional faces.

Our findings on recognition accuracy across facial expressions in the current study are generally consistent with those in prior research on emotional expression categorization showing highest recognition accuracy for happy expressions (Beaudry et al., 2014). However, our results showing that recognition accuracy of fear was better than for disgust, is contrary to a previous study (Calvo et al., 2018), which may be explained in terms of the cultural differences of the participants. We did not, however, find any response latency differences for the different expressions. Previous meta-analyses support a female advantage in decoding expression (i.e., happiness, anger, fear, disgust) which is related to greater attention to the eyes (Hall et al., 2010), however, we found no evidence for this sex difference.

While some previous studies have reported a general advantage in recognizing faces and face expressions of own- as opposed to other races (Sporer, 2001; Elfenbein and Ambady, 2002) we only found clear evidence for this with sad expression faces. Indeed, on the contrary there was a significant other-race bias for recognizing neutral, happy, fearful, and disgusted faces with no race-associated difference for recognizing angry faces. Interestingly, our Chinese participants made significantly more errors classifying neutral, happy, fear, and disgusted Asian expressions as sad, in comparison with the same White expressions. Sadness serves as an adaptive function to strengthen and sustain social bonds, especially in interpersonal relationships (Gray et al., 2011) which may underlie the observed own race bias for recognizing sad expression faces. Indeed, the fact that subjects were more likely to identify Asian than White neutral expression faces as sad tends to suggest that they have an overall greater sensitivity toward identifying individuals of their own race who may be feeling sad and in need of comfort, which further supports evidence for a greater tendency to attribute negative emotion to neutral faces of own and other races (Hu et al., 2017). Understanding and sharing other’s sadness, especially from one’s own race, may be a potential mechanism for racial bias in social behaviors due to it enhancing motivation for helping in-group members (Han, 2018).

More intense facial expressions tend to be recognized more accurately (Bänziger et al., 2009) and our finding that subjects rated the intensity and arousal of some White face expressions (i.e., anger, disgust, and fear) higher than Asian ones may have also contributed to the observed other-race bias for fearful and disgusted expressions, although not for happy, sad or neutral ones where ratings were equivalent or in the reverse direction. However, we found no correlations between recognition accuracy and intensity and arousal ratings for any White or Asian face expressions. It seems unlikely therefore that the greater intensity and arousal ratings seen for some White face expressions contributed significantly to greater recognition accuracy.

Interestingly, our pilot experiment demonstrated similar face expression recognition accuracy and intensity ratings for the Asian and White stimuli, indicating reasonable matching, however, in the main experiment we did find significant differences. Possibly this may be due to the larger number of subjects included in the main experiment, but it might also reflect the fact that subjects in the main experiment were exposed to the face stimuli twice rather than on a single occasion (once during eye-tracking and then again during behavioral ratings) and were also more strongly motivated by being required to make decisions as fast and accurately as possible as opposed to only being accurate. This may suggest that own- and other-race biases could be dependent to some extent on subject motivation and task difficulty.

Previous studies have indicated that the eye and the mouth regions, as well as the nose for specific expressions in some cases, are typically the most expressive sources (Calvo et al., 2018). In the current study, eye-tracking findings showed that subjects spent a greater proportion of time viewing the eye region across all expressions, followed by the nose and the mouth, similar to a previous cross-cultural study including East Asian participants (Jack et al., 2009). Another study has also reported that Asians spend more time viewing the eyes rather than the mouth region for accurate facial expression categorization, whereas White subjects pay more attention to the mouth (Yuki et al., 2007). Our results found that subjects viewed the eye region of Asian emotional, but not neutral, faces significantly more than White ones and also the nose region of sad Asian faces. This would support the pattern of eye-gaze toward own-race emotional faces being different to that for other-race ones. This difference was most notable during the later phases of viewing the different face expressions (from 1,000 to 3,000 ms) and could possibly reflect the point at which EEG studies have shown a late positive potential is evoked which is thought to indicate increased elaborative processing of emotional stimuli (Weinberg and Hajcak, 2011). However, this did not lead to increased recognition accuracy, and indeed there were no significant correlations between time spent gazing at the eye, nose, or mouth regions and emotion recognition accuracy for either Asian or White face emotions. However, it should be noted that accuracy judgments in our paradigm were made following a second subsequent presentation of all the facial stimuli and not when eye-tracking measures were actually recorded.

Consistent with previous studies, pupil sizes were larger when processing other-race (White) than own-race (Chinese) faces, especially when fixating the eyes and mouth regions. Increased pupil sizes may suggest greater cognitive load involved in processing other-race faces (Goldinger et al., 2009; Hu et al., 2017). Given that pupil sizes were measured during a free-viewing situation in which subjects were not instructed to discriminate between emotions intentionally, we can infer that they exerted more cognitive effort automatically when processing other-race faces. Pupil diameter is also associated with autonomic arousal (Bradley et al., 2008) which may reflect increased arousal ratings subjects gave for White angry, disgust and fearful faces, although there were no behavioral arousal differences found for happy, neutral, and sad White face expressions.

There are some limitations to the current study. Firstly, we have used subjects from a single culture in this eye-tracking study due to an ethnic diversity limitation and similar to many previous studies (Fu et al., 2012; Zhou et al., 2018; Hughes et al., 2019; Miao et al., 2020; Trawinski et al., 2020) reporting evidence for or against an own-race bias effect. Indeed, a meta-analysis of own-race bias in face emotion recognition has reported statistically similar findings from balanced studies including subjects from several cultures compared to unbalanced ones including subjects from only a single culture (Elfenbein and Ambady, 2002). It would have been interesting to include both Chinese and White subject groups to investigate the contribution of culture to own-race bias and a future cross-cultural study is needed to address this. Secondly, arousal ratings across the White and Asian face stimuli sets for some emotional expressions differed and this may have influenced our findings to some extent, although not for neutral, happy, and sad expressions. No single face database has sufficient numbers of both White and Asian face expressions for eye-tracking tasks and many studies do use multiple face databases, so we reasonably matched the two databases through face standardization and independent rating and found no significant differences. Thirdly, we decided to employ a free viewing eye-tracking paradigm in the study to avoid possible influences of conscious emotional evaluation and the requirement of making motor responses. Subjects only made judgments of expression identification subsequently during a second exposure to all stimuli and this may have contributed to the lack of significant correlations found between eye-tracking measures and accuracy judgments. In addition, it may be interesting to consider conducting a face identity recognition task rather than emotion identification one as a behavioral measure in the future. Fourthly, we did not attempt to quantify the amount of experience subjects had with White faces to address the issue of whether own-race differences are contributed by differential familiarity, however, previous studies have found no evidence for such a familiarity effect (Wong et al., 2020). A final issue was that we could not rigorously control the extent of mouth-opening for some facial expressions, although we found no evidence for differences in time spent viewing the mouth for Asian compared with White faces. Given reported recognition accuracy differences for sad, fear, and happy faces when the mouth is open as opposed to closed we made sure that in our stimulus sets the number of faces where the mouth was open was similar for the Chinese and White faces with these expressions (Tottenham et al., 2009).

In summary the findings from the current study support an own-race bias effect for identifying sad expressions in Chinese subjects and more generally across all face emotions in terms of a greater amount of time spent viewing the eyes and reduced pupil size, the latter possibly indicative of a reduced cognitive load.

## Data Availability Statement

The datasets presented in this study can be found in online repositories. The names of the repository/repositories and accession number(s) can be found in the article/supplementary material.

## Ethics Statement

The studies involving human participants were reviewed and approved by the Ethics Committee of the University of Electronic Science and Technology of China. The patients/participants provided their written informed consent to participate in this study. Written informed consent was obtained from the individual(s) for the publication of any potentially identifiable images or data included in this article.

## Author Contributions

XM and KK designed the study. XM, MF, XZ, RW, and XX conducted the experiments. XM, WZ, and XS analyzed the data. XM drafted the manuscript. WZ, KK, BB, and ZG provided critical revisions. All authors contributed to the article and approved the submitted version.

## Conflict of Interest

The authors declare that the research was conducted in the absence of any commercial or financial relationships that could be construed as a potential conflict of interest.

## Publisher’s Note

All claims expressed in this article are solely those of the authors and do not necessarily represent those of their affiliated organizations, or those of the publisher, the editors and the reviewers. Any product that may be evaluated in this article, or claim that may be made by its manufacturer, is not guaranteed or endorsed by the publisher.
